# Utilization of Anabolic Implants and Individual Supplementation on Muscle Growth and Protein Turnover During Backgrounding of Beef Steers

**DOI:** 10.3390/ani15040513

**Published:** 2025-02-11

**Authors:** J. Luke Jacobs, Elizabeth Leonard, Nishanth Tharayil, Susan K. Duckett

**Affiliations:** 1 Department of Animal and Veterinary Sciences, Clemson University, Clemson, SC 29634, USA; jjacob9@clemson.edu; 2 Multi-User Analytical Laboratory, Department of Plant and Environmental Sciences, Clemson University, Clemson, SC 29634, USA; eleona2@clemson.edu (E.L.); ntharay@clemson.edu (N.T.)

**Keywords:** beef, implant, supplementation, 3-methylhistidine, ultrasound, growth, protein turnover

## Abstract

Weaned Angus-cross steers (n = 69; body weight [BW] = 233 ± 29 kg) were used for the following purposes: (1) develop an assay to measure 3-methylhistidine (3MH) in blood samples to monitor changes in protein turnover, and (2) evaluate the effects of an implant (Revalor-G, 40 mg of trenbolone acetate, 8 mg of estradiol) or an implant plus individual supplementation via SuperSmart Feeder (SSF; C-Lock Inc., Rapid City, SD, USA) on animal growth, muscle growth, and nitrogen retention patterns in backgrounded beef steers. Steers were blocked by weight, trained to precision supplementation via SSF, and then assigned to one of three treatments: (1) grazing only [G], (2) grazing with implant [GI], or (3) grazing with implant plus individual animal supplementation via SSF at 0.75% BW [GIS] for 56 d of grazing study. Steers grazed a cowpea and pearl millet mixture until d 42, when forage became limiting, and steers were transitioned to an oat and annual ryegrass baleage. Steers were weighed and blood was collected every 14 d to determine plasma urea nitrogen (PUN), creatinine (CREAT), and 3-methylhistidine (3MH) concentrations. Ultrasound measurements of ribeye area (REA) were collected on d 0, 28, and 56. Data were analyzed using the GLIMMIX with treatment, day, and two-way interaction in the model. A novel, robust method for the determination of 3MH was developed and validated. The development of this 3MH method allows us to monitor changes in protein turnover in cattle over time, a metric which is related to production efficiency. A treatment-by-day interaction (*p* = 0.0050) was observed for BW and REA. Steer BW did not differ (*p* > 0.05) on d 0, 14, or 28 between treatments; however, on d 42, GI and GIS steers had a greater (*p* < 0.01) BW than G steers. On d 56, BW was greater (*p* < 0.0001) for GIS than GI and G, which not differ (*p* > 0.05). Total BW gain during the 56 d study was greater (*p* < 0.001) by 54% for GIS compared to GI or G. For GI, the total BW gain was greater (*p* < 0.01) by 18% compared to G. On d 56, the REA of GI and GIS steers was greater (*p* < 0.001) than of G steers. Steers in the GI treatment group had a greater PUN and PUN–CREAT ratio concentrations than the GIS steers. Both G and GI steers had greater 3-methylhistidine (3MH) concentrations compared to GIS steers on d 28. Our results demonstrate that the supplementation of implanted steers improves growth and improves nitrogen utilization during backgrounding on forage diets. This research illustrates that animal and muscle growth may be limited in grazing animals implanted with combination implants (estrogenic and androgenic compounds) when supplementation is not included.

## Simple Summary

The use of growth-promoting implants in beef production improves efficiency and reduces environmental impact. During the backgrounding phase, the use of anabolic implants may not be as beneficial as during feedlot phases due dietary differences that may limit protein and/or energy for growth. Muscle protein turnover rates in growing cattle are associated with feed efficiency; however, methodologies to measure protein turnover during growth are needed to make these assessments. This research developed a new method for 3-methylhistidine to measure in vivo protein turnover and examined how supplementation altered growth response to implanting in background calves. Our results show that animal and muscle growth may be limited in grazing animals implanted with combination implants when supplementation is not included.

## 1. Introduction

Optimizing forage utilization in beef production is imperative to lower the usage of human-edible feedstuffs and improve the sustainability of ruminant livestock production [1]. About a third of the annual cereal grain harvest is used for livestock feed rather than human food [2,3]. Feed costs are the single largest expense for livestock producers and account for up to 60% of total production costs in drylot programs, whereas the utilization of forages can reduce feed costs but strategic supplementation may be needed to optimize growth [4]. Muscle growth depends both on the rate of protein synthesis and protein degradation. Estimates are that 15–25% of energy intake in livestock is used to replace the muscle protein that is degraded during turnover [5,6,7]. Anabolic implants, especially those containing trenbolone acetate, reduce protein degradation, decrease nitrogen excretion, improve average daily gains, and improve animal efficiency [8,9,10,11]. The utilization of growth-promoting implants results in consistent improvements in beef cattle efficiency and lead to reductions in environmental impacts resulting from beef cattle production by increasing animal efficiency and reducing both the number of cattle required and decreasing the time to slaughter [12,13]. During the backgrounding phase, the use of anabolic implants may not be as beneficial as during feedlot phases due to the higher NDF content, which may limit protein and/or energy for growth [14].

In southeast US, most forages have crude protein levels above the requirement for growing cattle but differences in muscle mass are not observed in steers finished on forages versus concentrates [15]. The conversion of nitrogen into body weight gain for feedlot cattle is estimated at 14% [16]; nitrogen that is not retained by the animal is excreted in urine or feces and contributes to losses of nitrogen into the environment, with a negative impact on sustainability. Synchrony of nutrient supply is important in grazing animals to match energy and nitrogen supplies for improved microbial efficiency and nitrogen utilization [17]. Supplementation on forage can reduce protein loss, which may reach 30–40% in some systems [18]. de Oliveira Lazzarotto et al. [19] reported that supplementation on temperate grass pasture during backgrounding increased live weight gains by 47%. Wright et al. [20] found that grazing legumes versus grasses increased the average daily gain (ADG) by 0.15 kg/d and corn grain supplementation by 0.29 kg/d in finishing steers. Many backgrounding systems for beef calves utilize a drylot concentrate feeding system. The utilization of high-quality forages is paramount for minimizing human edible feedstuff in livestock production to improve sustainability.

Reducing protein turnover in growing calves will enhance muscle accretion; however, improved methodologies to monitor changes in 3 MH in blood samples are needed to assess how nutrition and management alter protein turnover in beef calves. An analytical method for the quantification of methylhistidine in blood plasma was optimized to improve the turnaround time and sensitivity of analysis from previously published methods [21] and was evaluated for accuracy, linearity, range, sensitivity, specificity, and stability. Derivatization with 2,2,3,3,4,4,4-heptafluorobutyl chloroformate (HFBCF) was initially reported for the analysis of amino acids on GC-MS and has demonstrated advantages as a quick and simple method for improved resolution and sensitivity [22]. After preliminary comparisons between GC-MS and LC-MS/MS, due to the higher sensitivity and robustness, our method optimization focused on the analysis of the HFBCF-derivatized MHs using HPLC-MS/MS. Thus, the objectives of this study were as follows: (1) to develop an assay to measure 3-methylhistidine (3MH) in blood samples to monitor changes in protein turnover, and (2) to evaluate the effects of an implant (Revalor-G, 40 mg of trenbolone acetate, 8 mg of estradiol) or implant plus individual supplementation of commercial feed via SSF on animal growth, muscle growth, and nitrogen retention patterns in backgrounded beef steers. We hypothesize that muscle growth and nitrogen utilization in grazing cattle receiving implants are limited without supplementation.

## 2. Materials and Methods

All animal and experimental protocols were approved by Clemson University Institutional Animal Care and Use Committee (AUP2021-0044).

### 2.1. Method Development

An analytical method for the quantification of methylhistidine (MH) in blood plasma was optimized to improve the turnaround time and sensitivity of analysis from previously published methods [21] and was evaluated for accuracy, linearity, range, sensitivity, specificity, and stability. Derivatization with 2,2,3,3,4,4,4-heptafluorobutyl chloroformate (HFBCF) was initially reported for the analysis of amino acids on GC-MS and has demonstrated advantages as a quick and simple method for improved resolution and sensitivity [22]. After preliminary comparisons between GC-MS and LC-MS/MS, due to the higher sensitivity and robustness, our method optimization focused on analysis of the HFBCF-derivatized MHs using HPLC-MS/MS. 2,2,3,3,4,4,4-Heptafluorobutyl chloroformate (HFBCF) was purchased from Synquest Laboratories (Alachua, FL, USA). The chemical standards for 1-methyl-L-histidine (1MH) and 3-methyl-L-histidine (3MH) were acquired from Sigma-Aldrich (St. Louis, MO, USA) and the stable isotope-labeled standard for 1-methyl-d_3_-L-histidine (1-MH-d_3_) was purchased from CDN Isotopes (Quebec, QC, Canada). Mass spectrometry (MS)-grade water, acetonitrile, ammonium formate, formic acid, pyridine, and reagent-grade sodium hydroxide were purchased from Fisher Scientific (Waltham, MA, USA), and isooctane and sodium carbonate were purchased from Sigma-Aldrich.

All standard solutions and reagents were prepared in MS-grade water. Reagents, sodium hydroxide (1 M), and sodium carbonate (0.5 M) solutions were prepared in water and mixed to prepare the neutralizing buffer (1 M NaOH–0.5 M Na_2_CO_3_ at a ratio of 4:1 (*v*/*v*)). The catalyst medium consisted of 50 mM sodium carbonate–pyridine at a ratio of 3:1 (*v*/*v*). The HFBCF reagent was prepared as HFBCF–isooctane at a ratio of 1:3 (*v*/*v*). Standard solutions for 1MH and 3MH were prepared in water at 1250 nM, 625 nM, 312.5 nM, 156.2 nM, 78.1 nM, 39.0 nM, 19.5 nM, 9.7 nM, and 4.8 nM.

**Figure 1:**
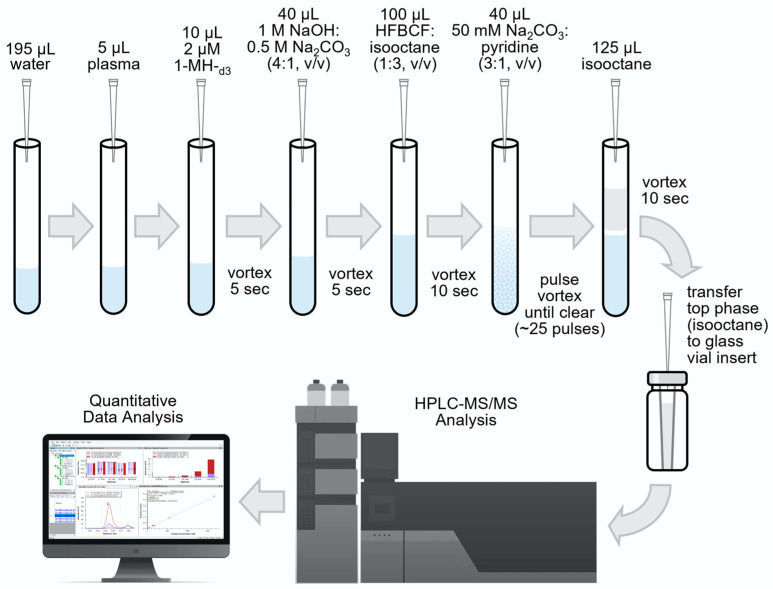
3-Methylhistidine schematic flow diagram.

1-Methylhistidine (1MH) and 3-methylhistidine (3MH) isomers from plasma were derivatized with 2,2,3,3,4,4,4-heptaflourobutyl chloroformate (HFBCF) for quantitative analysis on reversed-phase HPLC-MS/MS. Derivatization reactions were executed in 700 µL borosilicate glass culture tubes (6 × 50 mm) at room temperature. Further, 5 µL of plasma was added to 195 µL MS-grade water followed by 10 µL of 2 µM 1-MH-d_3_ as an internal standard. The mixture was vortexed at 3000 rpm for 5 s. This initial sample dilution step was modified for the derivatization of standards to add 200 µL of the calibration standard mixture (1MH and 3MH) to 10 µL of 2 µM 1-MH-d_3_, which was then vortexed at 3000 rpm for 5 s. Both samples and standards were derived as follows: 40 µL neutralizing buffer was added to the solution, vortexed at 3000 rpm for 5 s, and then 100 µL HFBCF reagent was added, vortexed at 3000 rpm for 10 s. The derivatization reaction was catalyzed with 40 µL of the catalyst medium (50 mM sodium carbonate–pyridine 3:1 [*v*/*v*]) and the solution was vortexed for 25 pulses until the top phase became clear. Following derivatization, 125 µL isooctane was added and vortexed at 3000 rpm for 5 s. An aliquot of 125 µL of the top isooctane phase was transferred to glass inserts for the analysis of 1 µL injection volumes on a high-pressure liquid chromatograph tandem mass spectrometer (HPLC-MS/MS; Figure 1).

Methylhistidine-HFBCF derivatives were separated on a Kinetex XB-C18 column of 2.6 µm 150 × 3 mm (Phenomenex, Torrance, CA, USA) using a Shimadzu LC-20AT pump coupled to a Shimadzu LCMS-8040 mass spectrometer (Nakagyo-ku, Kyoto, Japan). The HPLC solvent gradient employed mobile phases A (10 mM ammonium formate in MS-grade water with 0.062% formic acid) and B (0.1% formic acid in acetonitrile). The solvent gradient consisted of conditioning at 30% B at 0.0 min, followed by a linear gradient increase to 90% B by 2.0 min, a hold at 90% until 6.5 min, a decrease to 30% B at 6.7 min, and then hold at 30% B until 12.0 min, maintaining a flow rate of 0.35 mL/min throughout. The electrospray ionization source was operated in positive ionization mode with a capillary tube voltage of 4.5 kV, heat block temperature of 400 °C, desolvation line temperature of 250 °C, and nitrogen gas flow rates of 3 L/min and 15 L/min for nebulizing and drying gases, respectively. A Multiple Reaction Monitoring (MRM) method was employed for the quantification of analytes using the parameters in Table 1. The quantification of both 3- and 1-methylhistidine isomers was performed using the internal standard normalized peak area ratios. The internal standard recovery was above 70% and the limit of quantification of MH was estimated as 10 nM in the plasma matrix using the above method.

### 2.2. Method Validation

#### 2.2.1. Repeatability of Derivatization

The method’s precision for the HFBCF derivatization of methylhistidine in plasma was evaluated for intra- and inter-day precision. The repeatability (intra-day precision) of the derivative formation for methylhistidine and 1-MH-_d3_ was evaluated from five replicates of one plasma sample derivatized and analyzed within one day. Intermediate precision (inter-day precision) was determined from the derivatization and analysis of two replicates of one plasma sample per day, executed on three separate days. Precision was represented as the coefficient of variation (CV) of peak areas for the methylhistidine-HFBCF derivatives from HPLC-MS/MS analysis. The results are given in Table 2.

#### 2.2.2. Linearity of Response

The linearity of response was evaluated from linear regression models without weighting, using the internal standard normalized instrument response for each analyte as the measured variable on the y-axis and the concentration of the analyte on the x-axis. The internal standard normalized instrument response was calculated by dividing the peak area of each target analyte (3MH-HFBCF or 1MH-HFBCF) by the peak area of the stable isotope-labeled internal standard (1-MH-_d3_-HFBCF) within each data file. The linear ranges and regression coefficients (R^2^) are reported in Table 3.

#### 2.2.3. Limit of Quantification (LOQ)

The LOQ is defined as the lowest concentration of femtomole in the linear range that results in a signal-to-noise ratio (SNR) of at least 10:1 for the quantifier ion and at least 3:1 for the qualifier ion with acceptable ion ratios. The LOQ values for the HPLC-MS/MS analysis of methylhistidine-HFBCF derivatives are reported in Table 3.

#### 2.2.4. Specificity

**Figure 2:**
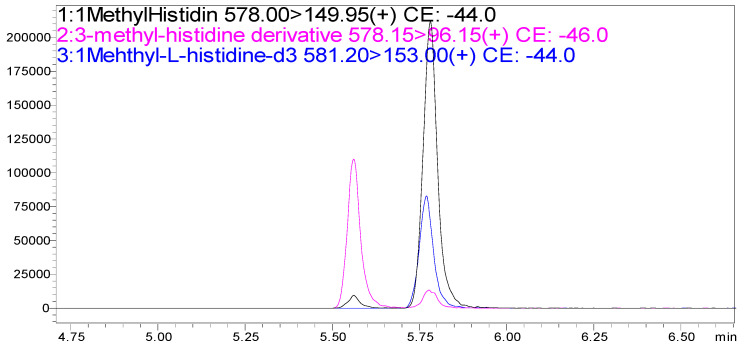
HPLC-MS/MS MRM chromatograms of a standard mixture of 185.2 fmol 3MH (pink), 185.2 fmol 1MH (black), and 100 fmol 1-MH-_d3_ (blue). The signal intensities for quantifier ion transitions are represented on the y-axis and the retention time in minutes on the x-axis.

**Figure 3:**
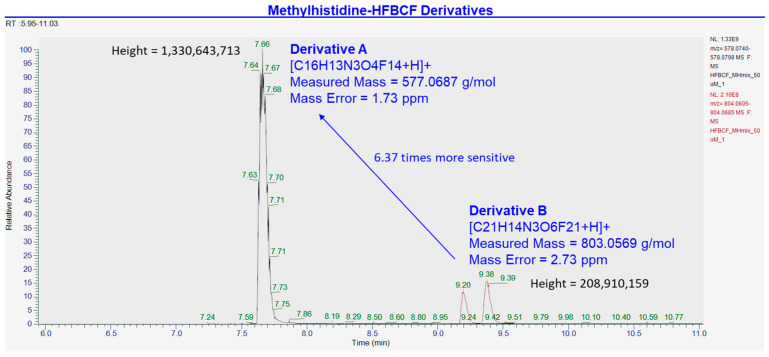
Comparison and selection of methylhistidine-HFBCF derivatives for quantification using HPLC-MS/MS high-resolution accurate mass analysis. Derivative A = molecular weight of 577.0687 g/mol and peak height of 1.33 × 10^6^; Derivative B = molecular weight of 803.0569 g/mol and peak height of 2.08 × 10^5^.

**Figure 4:**
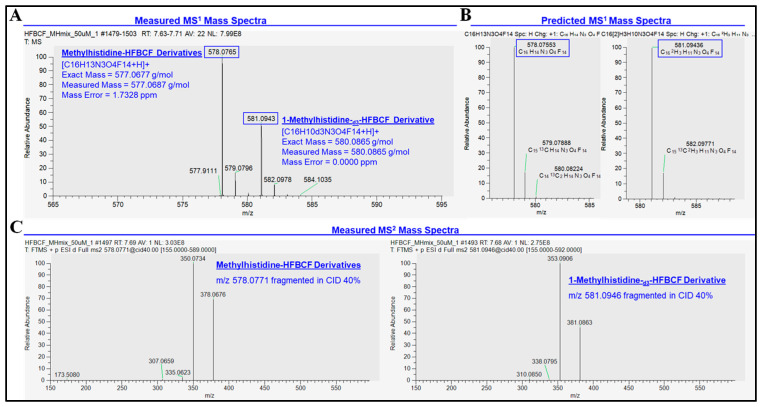
Confirmation of methylhistidine-HFBCF derivatives from (**A**) high-resolution accurate mass measurement of precursor ions in MS1 scan of a 50 μM standard mixture; (**B**) the predicted precursor ions of the methylhistidine-HFBCF derivatives; and (**C**) the MS2 fragmentation spectra of methylhistidine-HFBCF derivatives.

Specificity between the isomers, 3MH and 1MH, and the deuterated internal standard, 1-MH-_d3_, was established in two dimensions: (1) the chromatographic separation of isomers on a reversed-phase C18 column (Figure 2), and (2) mass separation with exact mass and distinctive MRM ion transitions (Table 1; Figure 2, Figure 3 and Figure 4). Detailed HPLC and MRM method parameters are described in Section 2.1.

##### HPLC Separation

The chromatographic separation of the HFBCF derivatives of the isomers 3MH and 1MH was carried out with a 12 min HPLC analysis consisting of a 6.5 min solvent gradient and a 5.5 min equilibration on a C18 reversed-phase column (Figure 2).

##### MRM and MS/MS Mass Separation

**Figure 5:**
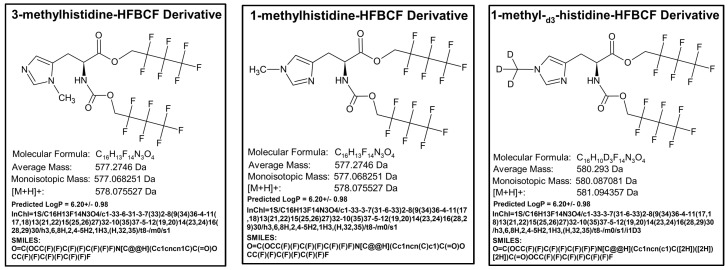
Chemical structures for the HFBCF derivatives of 3-methylhistidine, 1-methylhistidine, and 1-methyl-_d3_-histidine.

Distinction of co-eluting analytes, 1MH-HFBCF and 1-MH-_d3_-HFBCF, was based on the *m*/*z* due to the 3 amu mass difference imparted by the three deuterium replacements (1MH-HFBCF = 577.0677 g/mol; 1-MH-_d3_-HFBCF = 580.0865 g/mol). The characterization and selection of the HBCF derivatives used for quantification was performed using high-resolution accurate mass analysis (Figure 3, Figure 4 and Figure 5. The quantification of methylhistidines was performed using quantifier ions from MRM ion transitions, and confirmation using qualifier ions on a triple quadrupole mass spectrometer (Table 1, Figure 2). The detailed MRM method parameters are described in Section 2.1.

To verify the identity of the methylhistidine-HFBCF derivatives, a 50 μM standard mixture of methylhistidine-HFBCF derivatives was analyzed on a HPLC-MS/MS Orbitrap Fusion Tribrid mass spectrometer (Thermo Scientific, Waltham, MA, USA). Methylhistidine-HFBCF derivatives were ionized in a heated electrospray ionization source in positive ionization mode with 3.5 kV spray voltage, 300 °C ion transfer tube and vaporizer temperatures, and gas flow rates of 1, 10, and 35 arbitrary units for sweep, auxiliary, and sheath gas, respectively. The MS^1^ scan was collected in the orbitrap for a *m*/*z* 280–1400 mass range, with 60,000 (FWHM at *m*/*z* 400) resolution, 4 × 10^5^ automated gain control (AGC) target, 50 msec maximum injection time, and 35% lens. Mass fragmentation was carried out with a collision-induced dissociation (CID) of 40% and MS^2^ scan collected in the orbitrap with a resolution of 15,000 (FWHM at *m*/*z* 400), automatic scan range, 5 × 10^4^ AGC target, and 22 ms maximum injection time (Figure 3 and Figure 4).

Molecular structures, SMILES, InChI, and predicted LogP values of the methylhistidine-HFBCF derivatives 3MH-HFBCF, 1MH-HFBCF, and 1-MH-_d3_-HFBCF were created in ACD Labs ChemSketch (Freeware), 2024.1.2, file version C45E41.

#### 2.2.5. Stability of Derivatives

The stability of the methylhistidine-HFBCF derivatives in 20.57 nM standard mixture and in plasma samples after approximately 24 h of storage at 25 °C is reported in Table 4. Stability is represented as the percentage of the initial peak area and was calculated by dividing the peak areas measured at 24 h by the initial peak areas at 0 h and multiplying by 100%.

#### 2.2.6. Protein Precipitation (PP) in Blood Plasma

**Figure 6:**
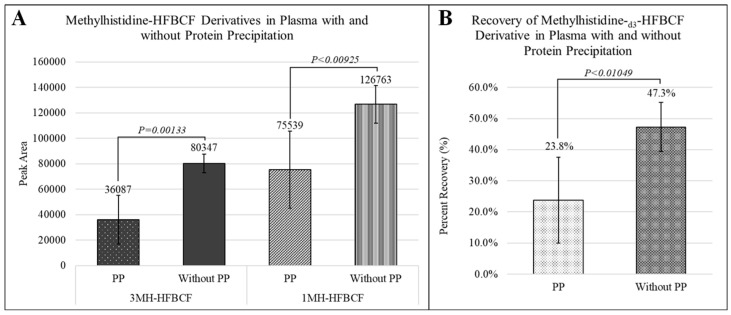
Effect of protein precipitation on (**A**) the yield of methylhistidine-HFBCF derivatives and (**B**) the recovery of 1-methylhistidine-_d3_-HFBCF in plasma. The stable isotope-labeled internal standard, 1-methylhistidine-_d3_-HFBCF, was added to the plasma samples before protein precipitation and derivatization with HFBCF. Five replicates per treatment group were included for statistical analysis. *p*-values were calculated from an unpaired two-tailed *t*-test.

Due to the relatively high composition of proteins in blood plasma, the effect of proteins on the derivatization efficiency was tested by treating plasma with and without PP prior to HFBCF derivatization. Here, 20 µL of plasma was spiked with two microliters of 40 μM 1-MH-_d3_ and then PP was carried out with 1:3 1-MH-_d3_ spiked plasma–acetonitrile (*v*/*v*), and plasma without PP was diluted 1:3 1-MH-_d3_ spiked plasma–water (*v*/*v*). Further, 20 µL of the 80 μL diluted plasma (PP and without PP) was transferred to 180 uL water in culture tubes and derivatized with HFBCF, as described in Section 2.1 without the addition of 10 μL 2 μM 1-MH-_d3_. This analysis was performed for five replicates of one plasma sample within the same day, *p*-values were calculated from unpaired two-tailed *t*-tests, and the results are reported in Figure 6.

### 2.3. Experimental Design

Angus-cross steers (3/4 Angus × ¼ Simmental; n = 69, BW = 231.8 ± 29.2 kg; 7 mo of age) were obtained from the Clemson University Piedmont Research and Extension Center. Steers were placed in a drylot for 13 d after weaning for training to an individual, automated supplement feeder, SuperSmart Feeder (SSF, C-Lock Inc., Rapid City, SD, USA). The SSF feeder had 4 feeding stations that the calves could consume the allotted supplement from. Once the allotted amount of supplement was consumed by each GIS calf, the SSF did not drop any more supplement that day. The gates on the SSF were raised from d 0 through d 3 to facilitate calf entry and were then lowered for the remainder of the training period to ensure individual access to the SSF. During the training period, steers were allotted 2.27 kg of a commercial supplement (Table 5) from the SSF daily, and 2.27 kg of the same feed in concrete feeders at 1400 h daily. Steers were allowed ad libitum access to clean water and bermudagrass hay (86% DM, 15.6% CP, 58.9% TDN, 78 Relative Feed Quality) throughout the duration of the training period. Additional information on training success rates with SSF for this study and others is available [23].

Following completion of the training period, steers (239.6 ± 28.9 kg) were assigned to one of three treatment groups (n = 23/trt) based on body weight (BW) and adaptation to the SSF. Treatment groups were as follows: (1) grazing only (G), (2) grazing with implant (GI), or (3) grazing with implant plus individual animal supplementation of the commercial supplement via SuperSmart Feeder at 0.75% BW (GIS). Steers that visited the SSF and consumed greater than 90% of the daily allotted feed during the training period were selected for use in the GIS treatment to ensure continual utilization of the SSF throughout the study.

### 2.4. Forage Management

Steers grazed 14.2 ha of a pearl millet/cowpea mixture (15.8% crude protein (CP), 49.6% total digestible nutrients (TDN), 111 relative feed value (RFV)). The pasture was divided into four paddocks of approximately 3.6 hectares using temporary fencing. All steers grazed the same paddock, and forage samples were collected weekly. Rotational grazing was managed to allow for ad libitum consumption of available forage. Steers grazed the pearl millet/cowpea paddocks until d 42, when forage availability became limited. Steers were then transitioned to a cool-season (oat/annual ryegrass; 9.1% CP, 63.6% TDN, 108 RFV) baleage from d 42 to d 56.

### 2.5. Weight Collection

Steers were gathered at 0700 h for each weight collection. For on-test and off-test weights, steers were weighed on two consecutive days and averaged. Steers were weighed every 14 d throughout the duration of the study.

### 2.6. Ultrasound Collection

Ultrasound measurements of fat thickness and ribeye area between the 12th and 13th rib of steers from all treatments were conducted on d 0, 28, and 56 of study. Ultrasound measurements were collected using an Aloka 500 V ultrasound unit (Corometrics Medical Systems, Wellingford, CT, USA), equipped with a 17 cm, 3.5 MHz linear probe. Images were interpreted using the Biosoft Toolbox (Biotronics, Inc., Ames, IA, USA). All images were collected and interpreted by an experienced technician to ensure consistency.

### 2.7. Administration of Implants

Steers assigned to GI or GIS treatment were administered a single Revalor-G implant (40 mg of trenbolone acetate and 8 mg estradiol; Merck & Co., Inc., Rahqay, NJ, USA) on d 0 of the study. Implants were administered subcutaneously in the middle third of the left ear after cleaning with a chlorohexidine solution. The implant gun needle was placed in a chlorohexidine solution following each use to ensure cleanliness and minimize contamination. After implantation and at weigh days, the left ear of each steer was palpated to confirm that the implant remained in the GI and GIS treatments.

### 2.8. Blood Collection

Blood samples were collected every 14 d throughout the duration of the study to monitor various blood metabolites and evaluate their relationship to nitrogen status and utilization. Blood samples were collected from the jugular vein using a vacutainer needle consisting of a 10 mL sodium heparin blood collection tube (BD Vacutainer, Becton, Dickinson and Company, Franklin Lakes, NJ, USA); vacutainer needle holder; and an 18-gauge, 3.81 cm multi-sample vacutainer needle (VWR, Batvavia, IL, USA). Immediately following sample collection, blood collection tubes were inverted and placed on ice until processing. Chilled blood samples were centrifuged at 2000× *g* for 20 min at 4.0 °C. Plasma was then collected, aliquoted, and stored at −80 °C until subsequent analyses.

### 2.9. Blood Metabolite Analysis

Plasma samples were analyzed for plasma urea nitrogen (PUN) and creatinine (CREAT) concentrations using a commercially available colorimetric detection kit (Arbor Assays, Ann Arbor, MI, USA), according to the manufacturer’s directions. Plasma urea nitrogen assays had an inter-assay coefficient of variation of 12.75% and an intra-assay coefficient of variation <6.5%. Creatinine assays had an inter-assay coefficient of variation of 8.11% and an intraassay coefficient of variance of <7.0%.

### 2.10. Statistical Analysis

Data were analyzed using the GLIMMIX procedure of SAS Version 9.4 (SAS Inst. Inc., Cary, NC, USA) as a completely randomized block design with treatment, time, and a two-way interaction in the model. Steer was the experimental unit for all analyses. If the interaction was significant, simple effect least square means were separated using a protected least significant difference test. If the interaction was non-significant, main effect least square means were separated using a protected least significant difference test. Significance was determined at (*p* < 0.05).

## 3. Results

### 3.1. Method Development

The optimized HPLC-MS/MS method for the quantification of methylhistidine-HFBCF derivatives demonstrated accuracy and precision, with intra- and inter- day CVs of <5% and <21%, respectively, in blood plasma (Table 2). Linearity was observed over nearly three orders of magnitude from 20.57 fmol to 1666.67 fmol from linear regression models with R^2^ values greater than 0.999 (Table 3). Method sensitivity was determined from limits of quantification (LOQ) in the low femtomole range: 20.57 fmol for both 3MH and 1MH (Table 3). Specificity between the methylhistidine isomers, 3MH and 1MH, and the deuterated internal standard, 1-MH-_d3_, was established through chromatographic separation on a reversed-phase C18 column and by mass separation with MRM ion transitions (Table 1; Figure 2). Two HFBCF derivatives of methylhistidine were detected with accurate mass (<2 ppm mass error) from the high-resolution accurate mass analysis on HPLC-MS/MS: Derivative A (577.0687 g/mol) and Derivative B (803.0569 g/mol). The peak height of Derivative A was observed to be 6.37-times higher than Derivative B in positive electrospray ionization (Figure 3). From the same analysis, the identity of the HFBCF derivatives was confirmed based on accurate mass, with mass errors of 1.73 ppm for methylhistidine-HFBCF (3- and 1- isomers) and 0.00 ppm for 1-MH-_d3_-HFBCF (Figure 4). The stability of the methylhistidine-HFBCF derivatives was observed for a 20.57 nM standard mixture and for a blood plasma sample stored at 25 °C for 24 h, with −10% to 12% maximum changes in the measured peak areas (Table 4).

The efficiency of deriving for plasma with and without pre-treatment with PP was evaluated. The average recovery of 1-MH-_d3_-HFCBF was significantly lower in PP-treated plasma (23.8%) than in plasma without PP (47.3%) (*p*-value = 0.01049). Further, the abundances of 3MH-HFBCF and 1MH-HFBCF were significantly lower in PP-treated plasma than in plasma without PP (*p*-value = 0.00133 for 3MH; *p*-value = 0.00925 for 1MH) (Figure 5). Without pre-treatment with PP, the precipitation of proteins in plasma was observed during the addition of isooctane after the derivatization reaction before analysis on HPLC-MS/MS.

### 3.2. Steer Performance

**Figure 7:**
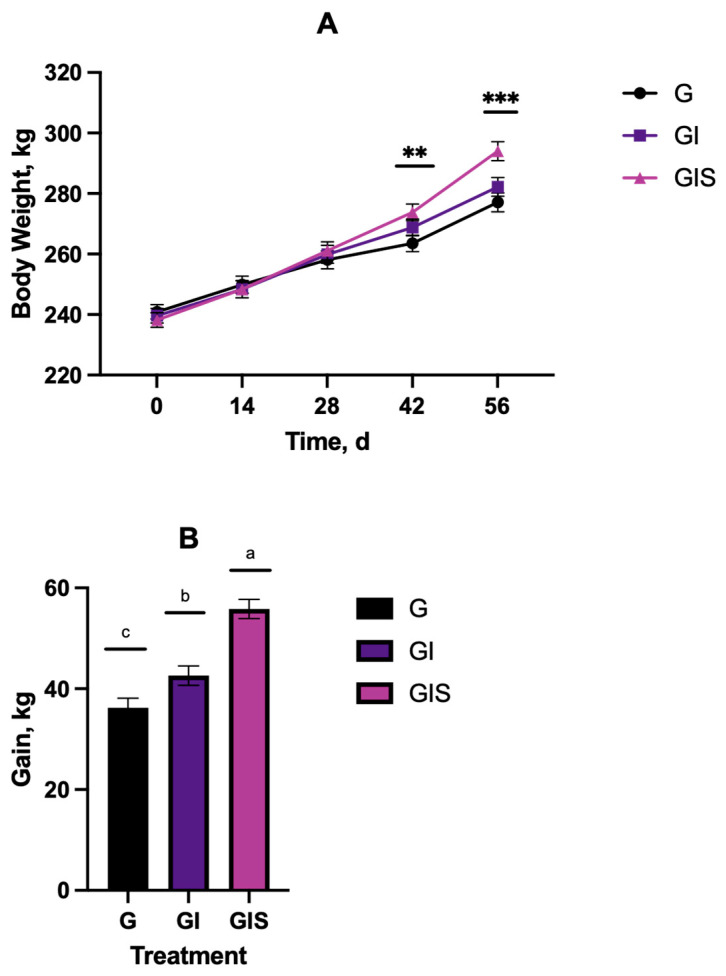
Effects of Revalor-G implant during backgrounding with (GIS) or without (GI) precision supplementation on body weight (**A**) or gain (**B**) compared to grazing only (G). ** Denotes significance at *p* < 0.01; *** *p* < 0.001; ^a, b, c^ Means with uncommon superscripts differ (*p* < 0.01).

A treatment-by-day interaction (*p* = 0.0050) was observed for body weight (BW) throughout the duration of the study (Figure 7). Steer weight did not differ (*p* > 0.05) on d 0, 14, and 28 between treatment groups. On d 42, BW was greater (*p* < 0.01) for the GI and GIS steers than the G steers. On day 56, BW was greater (*p* < 0.0001) for the GIS than GI and G, which not differ (*p* > 0.05). The total BW gain during the 56 d study was greater (*p* < 0.001) by 54% for the GIS steers compared to GI or G. For the GI steers, the total BW gain was greater (*p* < 0.01) by 18% compared to G.

### 3.3. Muscle Growth

**Figure 8:**
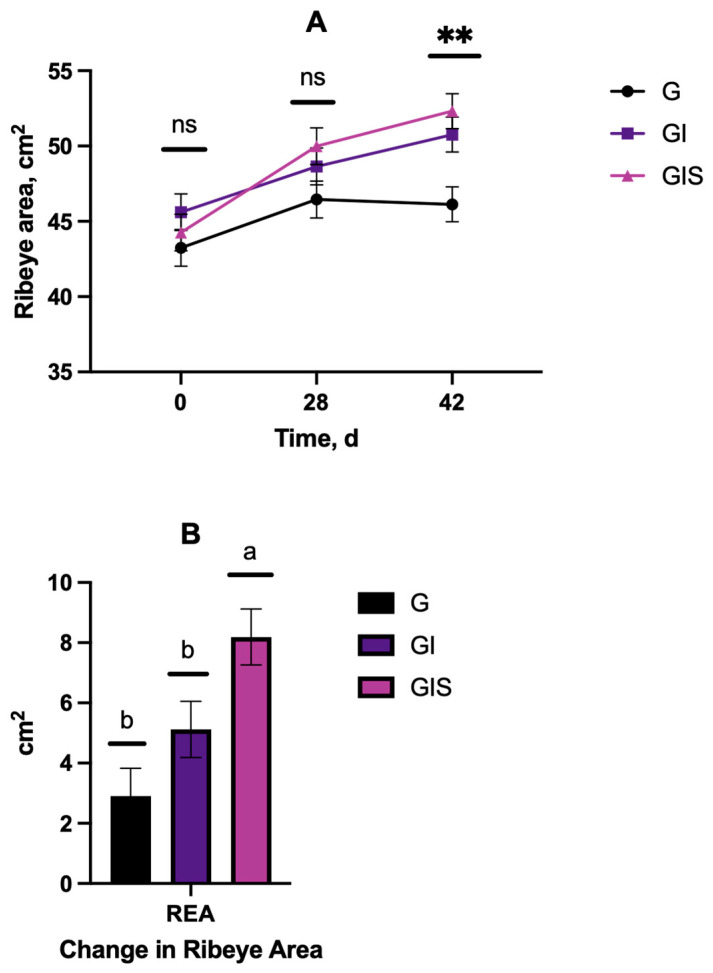
Effects of Revalor-G implant during backgrounding with (GIS) or without (GI) precision supplementation on ribeye area (**A**) or change in ribeye area (**B**) compared to grazing only (G). Denotes significance at ** *p* < 0.01; ^a, b^ Means with uncommon superscripts differ (*p* < 0.01).

A treatment-by-day interaction (*p* = 0.0007) was observed for ribeye area (REA; Figure 8 on d 0 and 28, no differences (*p* > 0.05)) between treatments. On d 56, the REA of the GI and GIS steers was greater (*p* < 0.001) than of the G steers. When ribeye area was normalized per unit body weight, there was no treatment-by-day interaction (*p* > 0.05); however, both treatment (*p* = 0.043) and day (*p* < 0.0001) differences were observed. The overall increase in REA during this 56 d study was 40% for GIS, which was greater (*p* < 0.001) than for the G and GI treatments.

### 3.4. Nitrogen Utilization

**Figure 9:**
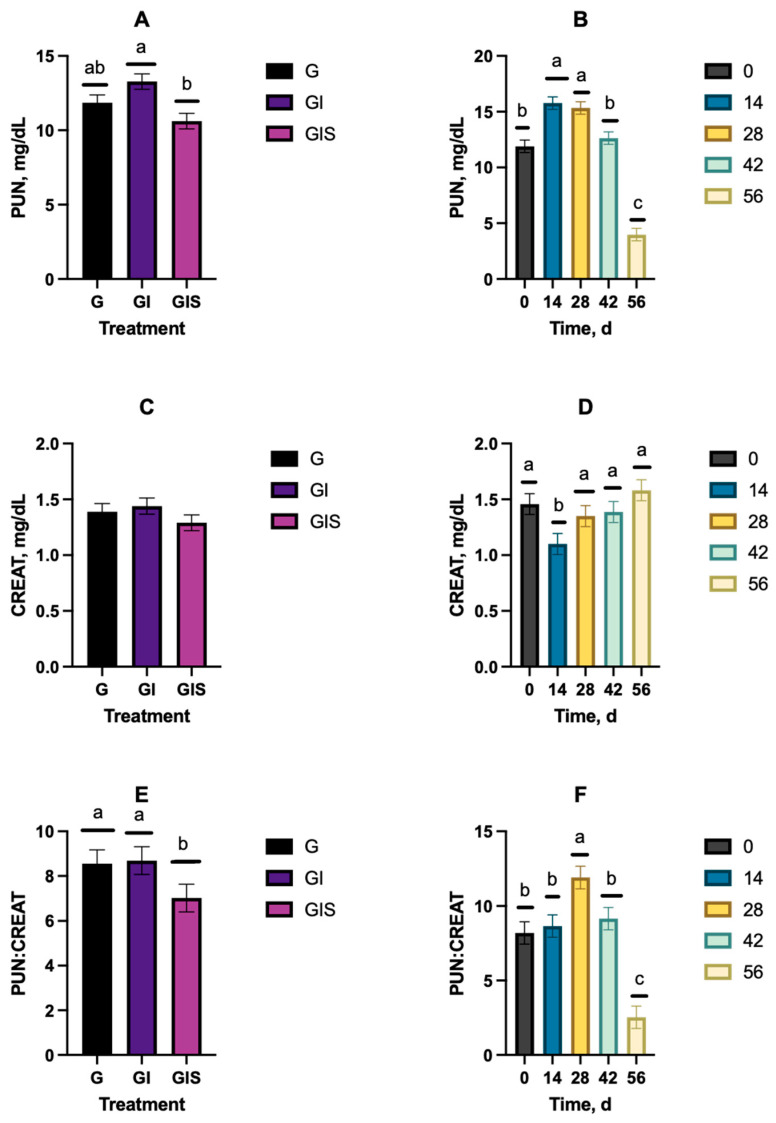
Changes in plasma urea nitrogen concentration (**A**,**B**), plasma creatinine concentration (**C**,**D**), and the ratio of plasma urea nitrogen to creatinine (**E**,**F**) during the backgrounding study. Treatment groups include grazing only (G), grazing with implant (GI), and grazing with implant and individual animal supplementation (GIS). ^a, b, c^ Means with uncommon superscripts differ (*p* < 0.05).

Plasma urea nitrogen concentration (PUN) differed by treatment (*p* = 0.0038; Figure 9A) and day (*p* < 0.0001; Figure 9B), but no treatment-by-day interaction was observed (*p* = 0.35). Steers in the GI treatment had a greater PUN concentration than the GIS steers, with G steers being intermediate (Figure 9A). Plasma urea nitrogen concentrations were greater (*p* < 0.001) on d 14 and 28 than d 0 and d 42, which were greater (*p* < 0.05) than on d 56. No treatment differences or treatment-by-day interaction (*p* > 0.05) were observed for CREAT concentrations throughout the study (Figure 9C); however, day was significant (*p* < 0.0001), with CREAT concentrations being lower (*p* < 0.05) on d 14 than all other time points (Figure 9D). Young and Munro (1978) reported the use of CREAT as an adjustment that can be utilized to compare the excretion of other metabolites to a similar apparent muscle mass. When evaluating PUN–CREAT ratios, there was no treatment-by-day interaction (*p* = 0.34); however, differences in both treatment (*p* = 0.011) and day (*p* < 0.0001) were observed. The G and GI steers had greater PUN–CREAT ratios compared to the GIS steers, but the G and GI steers did not differ from one another (Figure 9E). The PUN–CREAT ratio was highest (*p* < 0.05) on d 28 and lowest (*p* < 0.05) on d 56 (Figure 9F).

### 3.5. Protein Turnover

**Figure 10:**
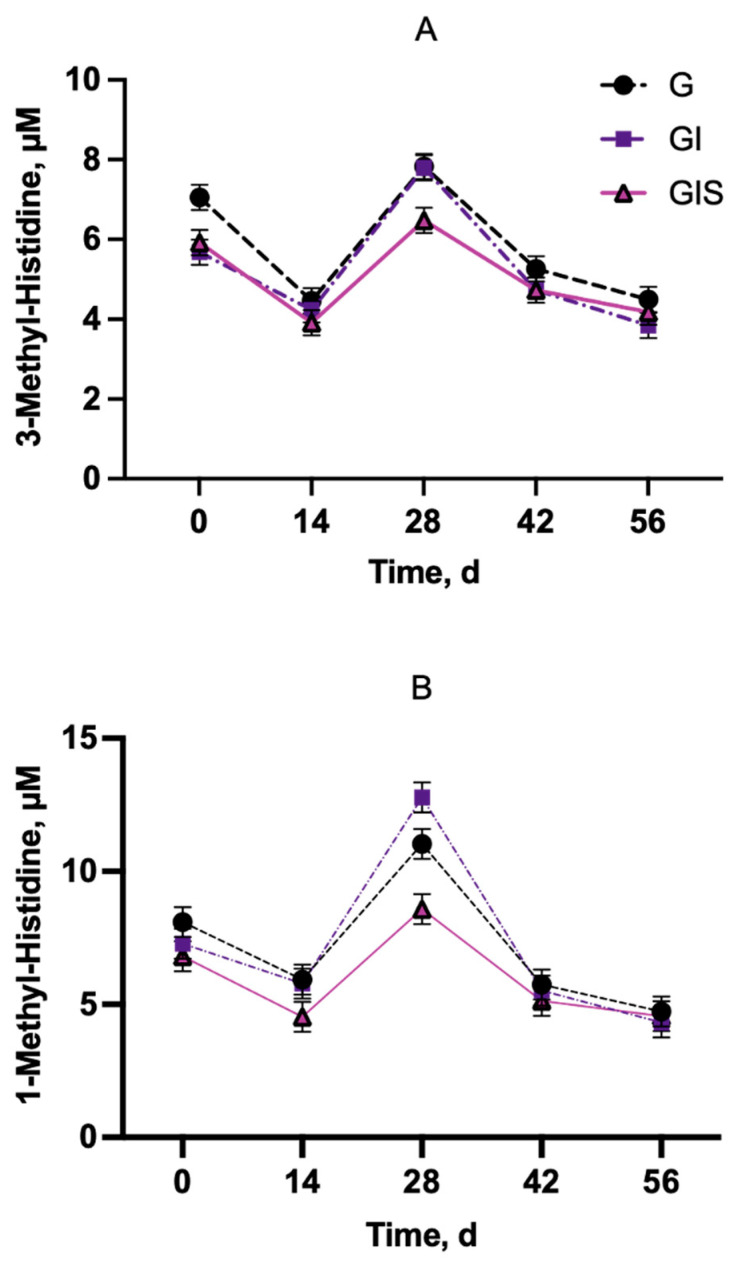
Effects of Revalor-G implant with (GIS) or without (GI) precision supplementation on plasma 3-methylhistidine (**A**) and 1-methylhistidine (**B**) concentrations compared to grazing only (G).

The average quantified concentrations of 3MH and 1MH were 5.38 μM and 6.72 μM, respectively, in beef steer blood plasma at 0, 14, 28, 42, and 56 d timepoints (Figure 10), and they are comparable to previous reports in dairy cows [21]. The average recovery of 1-MH-_d3_ in plasma samples was 71%, with a CV of 14%.

In the current study, a treatment-by-day interaction was observed (*p* = 0.0024) for 3MH concentration. On d 0, the G steers had a greater 3MH concentration than both the GI and GIS steers (Figure 10A). No differences between treatment groups were observed on d 14, but both the G and GI steers had greater 3MH concentrations compared to the GIS steers on d 28. No differences were observed between treatment groups on d 42 or 56 of study. A treatment-by-day interaction (*p* < 0.0001) was observed for the plasma 1MH concentration (Figure 10B). No difference in 1MH concentration was observed for days 0, 14, 42, or 56. On d 28, the GI steers had the highest 1MH concentration and the GIS steers had the lowest 1MH concentration.

### 3.6. Supplement Cost and Conversion

The average total supplement consumption of the GIS steers was 92.24 ± 1.56 kg, with an average daily supplement intake of 1.61 ± 0.03 kg (Table 6). The GIS steers exhibited an average frequency of SSF utilization of 89.04 ± 6.09%. The gain-to-supplement ratio observed in the GIS steers was 0.61 ± 0.02 kg gain to kg supplement. The net return per steer was USD 16.41 for GI and USD 30.53 for GIS.

## 4. Discussion

The method for the quantification of methylhistidine-HFBCF derivatives in plasma using HPLC-MS/MS was evaluated for accuracy, linearity, range, sensitivity, specificity, and stability. Compared to previously reported methods for quantifying methylhistidines in blood plasma, the optimized method showed significant improvements in analysis time and sensitivity [21,22].

The time of execution of the HFBCF derivatization reaction was approximately 5 min per plasma sample, blank, or standard. Methylhistidine-HFBCF derivatives, in standards and plasma, were shown to be stable for at least 24 h at room temperature (25 °C), and, with a 12 min HPLC-MS/MS analysis run time, approximately 120 injections could be made per day. The HFBCF derivatization of methylhistidine in plasma was shown to be efficient without the need for pre-treatment with protein precipitation. Even though the protein precipitation step was relatively detrimental to reproducibility in our analysis workflow, analyses that require a higher volume of plasma sample (either due to a lower sensitivity or a lower concentration of methylhistidines in samples) should adhere to protein precipitation before HFBCF derivatization. Similarly, even though not tested in this study, dry down of the HFBCF derivatives in the isooctane phase and subsequent reconstitution of the derivatives in acetonitrile or similar reversed-phase organic solvent could be tested for better instrument compatibility.

The sensitivity was improved to accurately quantify methylhistidine in plasma in the femtomole range, while previous methods report ranges in picomoles [21,22]. Due to its higher intensity in positive electrospray ionization, the methylhistidine-HFBCF derivative with a mass of 577.066 g/mol was selected for quantification over the derivative with a mass of 803.056 g/mol. However, depending on the instrument methods utilized, alternative HFBCF derivatives could be evaluated for quantification.

Along with the advantages that this method offers for improving analytical time and sensitivity, the use of a stable isotope labeled internal standard, 1-MH-_d3_, allowed for increased accuracy by normalizing concentrations for losses that may occur during sample preparation or from matrix effects during HPLC-MS/MS analysis. Additionally, this method could be valuable when sample volume is limited, as it requires extremely low sample volumes of five microliters plasma. Overall, compared to the previous method [21], the optimized method for the quantification of methylhistidine-HFBCF derivatives using HPLC-MS/MS resulted in a high-throughput method, with 1000 times higher sensitivity and 50 times lower sample volume.

Precision supplementation using the SSF in a pasture setting allowed for the individual supplementation of implanted steers at 0.75% BW for the GIS treatment. Supplementation of grazing cattle at <1% of BW has been shown to enhance performance [20,24,25], increase stocking rate [26], and improve carcass quality [27,28]. The results show that the precision supplementation of implanted steers (GIS) improved the average daily gain by 31.5% and 55.5% compared to implant only or grazing only steers, respectively. Implanting alone also increased body weight gain only by 18.6 over grazing, but not to the level of implanting with supplementation. Reinhardt and Thomson [29] reported 5% and 14% improvements in average daily gain with implantation during the suckling and stocker phases of beef production, respectively. They also showed that added weight gain with the implanting of grazing cattle was dependent upon the available nutrients and length of time on pasture. Bayliff et al. [30] implanted steers during the winter wheat stocker period with Revalor-G and found that implanting increased the average daily gain by 14.9% over non-implanted conditions. Berthiaume et al. [31] found that implanting with Revalor-G during the growing phase of beef production improved the ADG by 13% but that soybean meal supplementation (4 or 8% of the diet) had only minor effects on ADG. Cleale et al. [32] reported a 17.2% improvement in ADG in steers implanted with estradiol benzoate (21 mg) and trenbolone acetate (150 mg) pellets. These increased gains from growth-promoting implants provide potential economic incentives to encourage producers to utilize supplementation in addition to growth-promoting technologies [33]. Supplementation increases input costs, but it can also increase animal growth and value [34,35]. Overall, these results suggest that in order to achieve the optimal benefits of implants in backgrounding cattle on pasture, additional supplementation is needed. The additional weight gain from implanting and supplementation resulted in greater returns to the producer.

Skeletal muscle is an economically relevant tissue in livestock production and USDA muscle scores that influences cattle value when selling after the backgrounding/stocker phase of production [36]. Anabolic implants are known to increase ribeye area and muscle mass in feedlot cattle [8,9,37]. However, changes in muscle mass during the backgrounding or stockering phases of production have not been studied as widely. The use of ultrasound allowed us to monitor these changes in each animal over time during this phase of production. In this study, the ribeye area increased by 8.2 cm^2^ in steers that received an anabolic implant and precision supplementation, whereas the control and implant only steers increased by 2.9 and 5.1 cm^2^, respectively. These results also suggest that to optimize implant benefits during backgrounding, supplementation is needed to enhance growth and muscle mass in grazing steers. When ribeye size was normalized by body weight, there were no differences between the treatments, which indicates that the increase in muscle mass was proportional to the increase in body weight observed with precision supplementation and implant usage in this study.

Plasma urea nitrogen provides a point-in-time indicator of the protein–energy balance in beef cattle diets and could be used to further fine-tune diets or identify potential shortcomings in nutritional management programs [38,39]. It is also generally assumed that plasma urea nitrogen concentrations are equivalent to blood urea nitrogen concentrations because urea diffuses freely into and out of blood cells [39]. The use of growth-promoting implants has been shown to be associated with reduced blood urea nitrogen levels [40,41,42]. Our results indicate that GIS treatment reduced PUN and the PUN–CREAT ratio throughout the study compared to GI steers. Pate et al. [43] reported that blood urea nitrogen concentrations greater than 10 mg/dL are indicative of an adequate soluble intake protein relative to digestible energy intake. Using this reported threshold, steers of all treatment groups in the current study appeared to have adequate access to ample nutrients until d 56, when forage mass became limiting and steers were transitioned to a cool-season baleage. Hammond et al. [44] reported positive responses to energy supplementation of both steers and heifers grazing warm-season grass pastures when blood urea nitrogen concentrations were between 9.6 and 17.6 mg/dL. The results of the current study support the results reported by Hammond et al. [44], who reported that additional body weight gain was observed when supplemental energy was provided. Overall, the PUN–CREAT ratios were highest on d 28 and lowest on d 56. Creatinine is derived from the catabolism of creatine and phosphocreatine contained in the muscle and provides a measure of skeletal muscle mass [45,46]. Creatinine concentrations were lower on d 14 compared to all other time points. Treatment did not alter the creatinine concentrations in this study. These results suggest that supplementation with implantation allows for a more efficient use of available nitrogen and decreases nitrogen losses in the form of urea nitrogen compared to implantation alone in grazing steers. Clemmons et al. [47] examined the relationships between residual feed intake (RFI) and blood parameters. They found that a lower blood urea nitrogen-to-urea ratio was associated with low RFI, and that BUN measurements may be key indicators of feed efficiency.

3-Methylhistidine (3MH) is a breakdown product of actin and certain species of myosin heavy chain that occurs during post-translational modification [48]. 3-Methylhistidine is released and is not recycled for protein synthesis [49] or metabolized oxidatively [50] in beef cattle. The excretion of 3MH occurs in the urine and can serve as a marker of muscle protein degradation [51]. Harris and Milne [50] first validated that 3MH is quantitatively excreted in urine and is not reincorporated into myofibrillar protein in cattle. The determination of the 24 h urinary excretion of N^τ^-MH to measure muscle protein degradation has been used in cattle [52,53]. However, there is still debate regarding the contribution of 3MH excretion from tissues other than skeletal muscle and there may be a better indicator of total body muscle breakdown [45,46]. The collection of 24 h urine in grazing conditions is challenging; therefore, we examined plasma 3MH concentrations during the time course of this study to examine changes in protein degradation. Houweling et al. [21] reported the use of plasma 3MH to monitor muscle breakdown in dairy cows. The concentrations of 3MH observed in steers from the current study are similar to the 3MH concentrations of lactating dairy cows reported by Houweling et al. [21]. The concentrations of 3MH were highest on d 28, and the GIS steers showed reduced 3MH concentrations on d 28 compared to G and GI. Estimates are that 15–25% of the energy intake of cattle is used to replace muscle protein that is degraded during turnover [5,6,7]. The reductions observed in 3MH for GIS at this time frame preceded the increased body weight gain, indicating that reduced energy going to replacing degraded proteins may be responsible for the availability of greater energy for growth than maintenance. Combination (estrogenic and androgenic compounds) anabolic implants increase fractional accretion rates and fractional synthesis rates to enhance skeletal muscle protein accretion [7]. Johnson and Chung [54] reported that increases in protein deposition with combination implants are usually observed within the first 40 d after implantation. Due to its high metabolic cost, reductions in protein turnover rate are associated with residual feed intake (RFI) variation in cattle [45,55,56,57]. Guarnida-Lopez et al. [58] reported an association between RFI and fractional degradation rates (FDRs) in skeletal muscle, but that this response was diet-dependent with lower FDRs seen in low-RFI bulls fed corn silage but not grass silage.

1-Methylhistidine (1MH) or anserine is a dipeptide that is present in the muscle of many animals, including cattle [59]. Houweling et al. [21] reported both 1MH and 3MH values were similar, indicating that care should be taken to separate these two histidine metabolites to ensure the accurate quantification of 3MH concentrations when monitoring protein turnover. The concentrations of 1MH were highest on d 28, which coincides with 3MH concentrations and PUN–CREAT ratios. Steers on GIS treatment had a lower 1MH concentration on d 28, similar to 3MH concentrations.

## 5. Conclusions

The development of a rapid, sensitive method for the measurement of 3MH and 1MH in plasma samples provides us the opportunity to examine how nutritional or growth-promoting technologies can alter protein turnover in an animal over time. Our results demonstrate that the supplementation of implanted steers improves growth and improves nitrogen utilization during backgrounding on forage diets. This research illustrates that animal and muscle growth may be limited in grazing animals implanted with combination implants (estrogenic and androgenic compounds) when supplementation is not included.

## Figures and Tables

**Table 1:** MRM ion transitions for methylhistidine-HFBCF derivatives from HPLC-MS/MS analysis; quantifier ions appear in bold font.

Compound Name	Compound Name Abbreviation	Precursor Ion *m*/*z*, [M + H]^+^	Product Ion *m*/*z*, [M + H]^+^	Collision Energy (V)
3-methylhistidine-HFBCF	3-MH-HFBCF	578.1	**96.1**	−46
			378.0	−38
			335.0	−43
1-methylhistidine-HFBCF	1-MH-HFBCF	578.0	**149.9**	−44
			350.0	−32
			378.0	−31
1-methylhistidine-_d3_-HFBCF	1-MH-_d3_-HFBCF	581.2	**153.0**	−44
			353.0	−34
			381.0	−32

**Table 2:** Method’s precision for the HFBCF derivatization of blood plasma by intra- and inter-day precision. Precision was calculated as the coefficient of variation in peak areas for methylhistidine-HFBCF derivatives.

	Coefficient of Variation (%)		
	3MH-HFBCF	1MH-HFBCF	1MH-_d3_-HFBCF
Intra-day Precision in Plasma	4.8%	1.9%	1.4%
Inter-day Precision in Plasma	12.8%	20.2%	8.4%

**Table 3:** Limits of quantification (LOQ), linear range, and regression coefficients (R^2^) for methylhistidine-HFBCF derivatives in HPLC-MS/MS.

	3MH-HFBCF	1MH-HFBCF
LOQ (nM)	20.57	20.57
Range (nM)	20.57–1666.67	20.57–1666.67
R^2^	0.9994–0.9999	0.9999–1.0000

**Table 4:** Stability of methylhistidine-HFBCF derivatives in 20.57 nM standard mixtures and in plasma samples with storage at 25 °C.

		Percent of Initial Peak Area (%)		
	Storage Time (Hours)	3MH-HFBCF	1MH-HFBCF	1MH-_d3_-HFBCF
20.57 nM Standard Mixture	23.5	95%	90%	102%
	25.5	99%	95%	101%
Plasma Sample	24.5	94%	98%	112%

**Table 5:** Forage and supplement dry matter (DM), crude protein (CP), acid detergent fiber (ADF), neutral detergent fiber (NDF), total digestible nutrients (TDNs), ash, and relative feeding value (RFV) for this study. The commercial supplement was a CPC (Fountain Run, KY, USA) 14% developer with a proprietary mixture of roughage products, corn gluten pellets, processed grain by-products, steam flaked corn, calcium carbonate, and CPC premix.

	DM	CP	ADF	NDF	TDN	Ash	RFV
Hay	86.0	15.6	36.4	72.5	58.9	5.6	78
Supplement	89.5	20.6	19.5	40.2	75.3	6.0	-
Forage	27.4	17.9	35.0	53.3	56.2	6.4	111
Baleage	36.3	9.1	34.4	53.7	63.6	6.4	108

**Table 6:** Effect of implanting with (GIS) or without (GI) supplementation on cost of production and net return of steers compared to grazing only (G).

	G	GI	GIS
Implant cost, USD/hd	USD 0	USD 1.44	USD 1.44
Supplement intake, kg	0	0	92.24
Supplement cost ^a^, USD	0	0	USD 26.75
Final steer weight, kg	277.0	282.3	294.1
Steer average daily gain, kg/d	0.64	0.76	1.00
Supplement conversion (gain–supplement)	-	-	0.61
Steer price, USD/kg	USD 3.454	USD 3.454	USD 3.454
Steer value, USD/hd	USD 956.76	USD 975.06	USD 1015.48
Steer value over G, USD/hd	-	USD 18.30	USD 58.72
Implant + suppl. cost, USD/hd	-	USD 1.44	USD 28.19
Net return per steer, USD/hd	-	USD 16.41	USD 30.53

^a^ Supplement cost = USD 0.288/kg.

## Data Availability

The raw data supporting the conclusions of this article will be made available by the authors on request.

## Abbreviations

The following abbreviations are used in this manuscript:

1MH	1-methylhistidine
1MH-d3-HFBCF	1-methylhistidine-d3-HFBCF derivative
1MH-HFBCF	1-methylhistidine-HFBCF derivative
3MH	3-methylhistidine
3MH-HFBCF	3-methylhistidine-HFBCF derivative
ADG	Average daily gain
BW	Body weight
CREAT	Creatinine
G	Grazing only treatment
GI	Grazing plus anabolic implant treatment
GIS	Grazing plus anabolic implant and precision supplementation treatment
HFBCF	2,2,3,3,4,4,4-heptafluorobutyl chloroformate
PUN	Plasma urea nitrogen
REA	Ribeye area
SSF	SuperSmart Feeder
